# Evaluation of Antibacterial and Antifungal Properties of *Alchornea laxiflora* (Benth.) Pax. & Hoffman

**DOI:** 10.1155/2015/684839

**Published:** 2015-01-19

**Authors:** David A. Akinpelu, Emmanuel O. Abioye, Olayinka A. Aiyegoro, Oluseun F. Akinpelu, Anthony I. Okoh

**Affiliations:** ^1^SA-MRC Microbial Water Quality Monitoring Centre, University of Fort Hare, Alice 5700, South Africa; ^2^Applied and Environmental Microbiology Research Group, Department of Biochemistry and Microbiology, University of Fort Hare, Alice 5700, South Africa; ^3^Department of Microbiology, Obafemi Awolowo University, Ile Ife 234, Osun State, Nigeria; ^4^GI Microbiology and Biotechnology Unit, Agricultural Research Council, Animal Production Institute, Irene, Pretoria 0062, South Africa; ^5^Department of Biological Science, Faculty of Agriculture and Technology, North-West University, Mafikeng Campus, Mmabatho 2735, South Africa

## Abstract

*Alchornea laxiflora *leaf extract was tested against a range of microorganisms using standard microbiological methods for antimicrobial activities. The extract inhibited the growth of all the bacterial and 15 fungal isolates tested. The zones of inhibition exhibited against the test bacteria ranged between 12 mm and 24 mm and between 11 mm and 24 mm for the extract and the antibiotic streptomycin, respectively. The zones of inhibition observed against the fungal isolates by the extract ranged between 12 mm and 23 mm. The minimum inhibitory concentrations (MICs) and the minimum bactericidal concentrations (MBCs) exhibited by the extract against test bacteria ranged between 0.78 mg/mL–25 mg/mL and 1.56 mg/mL–25 mg/mL, respectively, while the MICs and minimum fungicidal concentrations (MFCs) values for the test fungi ranged between 8.75 mg/mL–35.00 mg/mL and 8.75 mg/mL–35.00 mg/L, respectively. The preliminary phytochemical screening of the extract revealed the presence of alkaloids, tannins, flavonoids, saponins, and reducing sugars as major phytoconstituents in the extract.* A. laxiflora *leaf extract is a potent source of antibacterial and antifungal compounds; further studies on the extract are ongoing in our laboratories to elucidate the probable mechanism(s) of action on bacteria and fungi found to be susceptible to the extract.

## 1. Introduction

Medicinal plants have been in existence since creation and these plants synthesize a wide variety of phytochemicals which include alkaloids, tannins, flavonoids, steroids, saponins, and phenols, which are all useful sources of medicine [1]. Also, medicinal plants have been proved to be effective in the treatment of infectious diseases with little or no side effects as experienced with synthetic drugs [2]. The biologically active components of plant extracts and essential oils are used in most of the pharmaceutical industries because of their antimicrobial, antifungal, and antiviral properties. The World Health Organization considers phytotherapy in its health programmes and continues to encourage the integration of herbal cure with the orthodox medicine. Infectious diseases caused by bacteria, fungi, and viruses are still in increase and they are still the major threat to public health. Efforts are still ongoing to search for new biologically active compounds from natural sources as new antimicrobial agent with a view to discovering new chemical structures which could overcome the multiple resistances developed by the pathogenic microbes toward available antimicrobial agents.

*Alchornea laxiflora* belongs to Euphorbiaceae family; it is a deciduous shrub and about 6–10 m high. It grows naturally in Nigeria, in DR Congo, in Ethiopia, and throughout East Africa to Zimbabwe [3]. The plant is monoecious having its male and female inflorescences on separate branches.* Alchornea laxiflora* is called “Opoto” among the Yoruba tribe in Nigeria. The leaf infusion of the plant is often used in folklore medicine as antimalarial [4]. The stem, especially the branches, is used in Nigeria as chewing sticks (local tooth brush) for cleaning teeth while the leaves are used to preserve kola nut and other perishable fruits and vegetables. Decoction of the leaves is usually administered to treat inflammatory and infectious diseases [5]. Oladunmoye and Kehinde [6] reported the use of* A. laxiflora* among the Yoruba tribe of Southwestern Nigeria for the treatment of poliomyelitis and measles. Other authors, Oloyede et al. [7], reported the antioxidant properties of the leaf extract of* A. laxiflora* in addition to its antimicrobial effects on four bacteria which are* Staphylococcus aureus*,* Escherichia coli*,* Bacillus subtilis*, and* Pseudomonas aeruginosa*. Farombi et al. [8] and fellow investigators also reported the antioxidant properties of* A. laxiflora* leaves and roots. The results obtained from their investigation indicate the presence of potent natural antioxidant which may be relevant in preservation of lipid food products. Antitoxicity, anticonvulsant, and sedative effects of the leaf extract of* A. laxiflora *in animal models have been reported by Esosa et al. [9]. The leaf extract has been reported to contain alkaloids, flavonoids, saponins, tannins, carbohydrates, cardioactive glycosides steroids, phenols, and reducing sugars as phytochemicals resident in the plant.

## 2. Materials and Methods

### 2.1. Plant Sample

*Alchornea laxiflora* fresh leaves were collected from Abeokuta, Ogun State, Nigeria, in the month of March 2014. The leaf was identified in the Herbarium of the Department of Botany, Obafemi Awolowo University, Ile Ife, Nigeria. The leaves were oven-dried at 40°C until a constant weight was observed. The dried leaves were milled into a fine powder and later stored in an air-tight bag until use.

### 2.2. Preparation of the Leaf Extract

Exactly 850 g of the powdered leaves of* A. laxiflora *was soaked in the mixture of methanol and sterile distilled water in ratio 3 : 2 (v/v) and left on the laboratory bench for four days with regular agitation. The solution was filtered and the supernatant collected was concentrated* in vacuo* to expel the organic solvent leaving aqueous part which was later lyophilized and 115 g yield was collected.

### 2.3. Preparation of Bacterial and Fungal Isolates Used for the Experiment

Bacterial isolates used for this study include typed cultures and isolates from surgical wound (SW), sepsis (SS), stool (ST), nasal cavity (NC), and environmental strains (ES):* Staphylococcus aureus* (NCIB 8588),* Staph. aureus* (SW),* Staph. aureus* (SS),* Staph. aureus* (NC),* Shigella* species (ST),* Escherichia coli* (ST),* Klebsiella pneumoniae* (NCIB 418),* K. pneumoniae* (SS),* Bacillus polymyxa* (ES),* Clostridium pyogenes* (ES),* Proteus vulgaris* (ES),* Pseudomonas aeruginosa* (ES),* Bacillus anthracis* (ES),* Micrococcus luteus* (NCIB 196),* Pseudomonas fluorescens* (NCIB 3756),* Bacillus cereus* (NCIB 6349),* Clostridium sporogenes* (NCIB 532),* Bacillus stearothermophilus* (NCIB 8222),* E. coli* (NCIB 86),* Bacillus subtilis* (NCIB 3610),* Enterococcus faecalis* (NCIB 775), and* Pseudomonas aeruginosa* (NCIB 950).

The fungal isolates used were* Aspergillus niger*,* Aspergillus fumigatus*,* Aspergillus glaucus*,* Aspergillus flavus*,* Fusarium* species,* Penicillium expansum*,* Alternaria* species,* Trichophyton interdigitale*,* Trichophyton mentagrophytes*,* Trichophyton rubrum*,* Penicillium camemberti*,* Trichoderma* species,* Scopulariopsis brevicaulis*,* Penicillium italicum*,* Candida albicans*, and* Candida pseudotropicalis*.

### 2.4. Phytochemical Assay of the Leaf Extract

The crude leaf extract of* A. laxiflora* was subjected to phytochemical assay using [10, 11] methods.

### 2.5. Determination of Antibiograms of* A. laxiflora* Leaf Extract, Standard Antibiotic and Antifungal Compounds

The antibiograms of the extract along with those of the antifungal and antibiotic were determined using agar-well diffusion methods by [12, 13] with little modification. The bacterial isolates were subcultured into nutrient broth before use while the fungal isolates were cultured in malt extract agar medium and left for seven days for proper sporulation before spores harvesting. The 18-hour-old bacterial culture was standardized using McFarland standard (10^6^ cfu/mL of 0.5 McFarland standard). The fungal spores were harvested after sporulation by pouring mixture of sterile glycerol and distilled water to the surface of the plate and scraping the spores with sterile glass rod. The spores were later standardized before use. One hundred microliters of each of the standardized bacterial and fungal suspension was evenly spread on Mueller-Hinton agar medium for bacteria and malt extract medium for the fungi using a sterile glass spreader. Sterile cork borer was used to bore holes into the agar medium allowing about 5 mm distance to the edge of the plate. The plates with bacteria cultures were treated with the solution of the extract at a final concentration of 25 mg/mL while those for fungi were treated with solution at 35 mg/mL. The plates were allowed to stand on the laboratory bench for one hour to allow for proper diffusion of the extract solution into the medium. The plates with bacteria culture were incubated at 37°C for 24 hours while those with fungi were incubated at 25°C for upward 96 hours after the plates were observed for zones of inhibition. The effect of the extract on bacteria was compared with that of streptomycin while that of fungi was compared with Nystatin both at a concentration of 1 mg/mL.

### 2.6. The Minimum Inhibitory Concentrations (MICs) of the Extract against the Test Organisms

The method described by [14] was used to determine the MIC of the extract against the test isolates. A twofold dilution of the extract was prepared and 2 mL aliquots of different concentrations of the solution were added to 18 mL of presterilised molten nutrient agar medium for bacteria and malt extract for fungi at 40°C. The final concentrations regimes of 0.78 mg/mL and 25.00 mg/mL were used against bacteria and 2.19 mg/mL and 35.00 mg/mL were used against fungi. The medium was poured into sterile Petri dishes and allowed to set. The plates were left on the laboratory bench for 24 hours to observe their sterility. The dry surface of the media was later streaked with standardized 18-hour-old bacteria culture while 0.1 mL of the standardized fungal spores was evenly spread out on malt extract medium. The plates inoculated with bacterial culture were incubated at 37°C for up to 72 hours and the plates with the test fungi were incubated at 25°C for 96 hours. These were later examined for the presence or absence of growth. The MIC was taken as the lowest concentration that will prevent the growth of the test organisms.

### 2.7. The Minimum Bactericidal Concentrations (MBCs) and Minimum Fungicidal Concentrations (MFCs) of the Extract against the Test Isolates

Assay for the MBCs/MFCs was determined using Olorundare et al. [15] with little modification. Samples were taken from plates with no visible growth in the MIC assay and subcultured onto freshly prepared nutrient agar plates for bacteria and malt extract medium for fungi. The plates subcultured with bacteria were incubated at 37°C for up to 72 hours while fungal plates were incubated at 25°C for up to 96 hours. The MBC or MFC was taken as the concentration of the extract that did not show any bacterial or fungal growth on fresh agar medium.

## 3. Results

The antimicrobial activities of* A. laxiflora* leaf extract were studied against some panel of bacterial and fungal isolates. The extract exhibited antibacterial and antifungal properties as shown in Tables 1 and 2.

The extract of* Alchornea laxiflora* at a concentration of 25 mg/mL inhibited the growth of all the test bacterial isolates, whereas the standard antibiotic (streptomycin) used as a positive control in this study inhibited 26 out of 39 isolates (Table 1). Also, the extract at concentration of 35 mg/mL inhibited the growth of 15 fungal isolates out of a total of 17 tested (Table 2). The zones of inhibition exhibited by the leaf extract against the test bacteria ranged between 12 mm and 24 mm; on the other hand, the range of zones of inhibition shown by streptomycin falls between 11 mm and 24 mm (Table 1), and the zones of inhibition observed against the fungal isolates ranged between 12 mm and 23 mm (Table 2).

The minimum inhibitory concentrations as well as the minimum bactericidal concentrations were determined. The MIC exhibited by the leaf extract of* A. laxiflora* against test bacteria ranged between 0.78 mg/mL and 25 mg/mL. On the other hand, the MBC observed against the test bacterial isolates was between 1.56 mg/mL and 25 mg/mL (Table 1).

The MICs and MFCs shown against the test fungi through the activity of* A. laxiflora* leaf extract ranged between 8.75 mg/mL and 35.00 mg/mL for MIC and 8.75 mg/mL and 35.00 mg/L for the MFC (Table 2).

The phytochemical assay of the leaf extract of* A. laxiflora* revealed the presence of alkaloids, tannins, flavonoids, saponins, and reducing sugars (Table 3).

## 4. Discussion

*Alchornea laxiflora* leaf extract exhibited appreciable antibacterial and antifungal activities against the panel of bacteria and fungi used in this study. The results obtained revealed the inhibition of both Gram-positive and Gram-negative bacteria while a wide range of fungi used were susceptible to the effects of the plant extract. This is an indication of broad spectrum activities exhibited by* A. laxiflora* leaf extract.

*Pseudomonas aeruginosa* known to be resistant to many antibiotics was susceptible to the effect of the extract. Also inhibited by the extract is* Staph. aureus*, a pathogen known to cause various diseases in humans and animals. Other pathogenic bacteria found to be inhibited by this extract include* E. coli*, causative agent of diarrhoea, urinary tract infections, and so forth, and* K. pneumoniae*, causative agent of pneumonia [16].

The susceptibilities of these pathogens to the leaf extract of* A. laxiflora* corroborate the use of this plant in folklore remedies for the infections caused by microbes and thus demonstrated the significant therapeutic potential of this plant.

The antimicrobial effects of the leaf extract of* A. laxiflora* on some fungi were also examined.* Aspergillus* species which were among the fungal isolates used and known to cause aspergillosis mainly bronchopulmonary aspergillosis were susceptible to this plant extract.

Dermatophytes such as* Trichophyton tonsurans*,* T. mentagrophytes*, and* T. interdigitale* studied were also inhibited by the leaf extract of* A. laxiflora*. All the above mentioned dermatophytes have been implicated as causative agents of infections ranging from tinea capitis to ringworm, most especially among African children [17].

The extract was able to inhibit the growth of these pathogenic dermatophytes which is an indication that extract from this plant is a good source of antifungal compound that can be employed in pharmaceutical industries for the formulation of potent antifungal drugs.

*A. laxiflora* leaf extract also inhibited the growth of* Alternaria* species in this study.* Alternaria *species is a plant pathogen capable of causing hay fever in humans leading to asthma [16]; thus, the extract may be a prototype for future drugs in the management of respiratory tract complications such as asthma.

*Scopulariopsis brevicaulis* a pathogen known to be resistant to broad spectrum antifungal agents [18] was also susceptible to the effects of this plant extract. This is an indication that* A. laxiflora* is a good source of antimicrobial compounds and thus supports its uses traditionally for the treatment of various infections caused by pathogens both fungi and bacteria. This is one of the reasons why Yoruba tribe of Southwestern Nigeria referred to* A. laxiflora* as “Gbogbonse.”

Our investigations on* A. laxiflora* revealed the abilities of this plant to inhibit or kill pathogens at lower concentration (though still in crude form) and thus could be used in preventing the establishment of infections caused by these pathogens. The results obtained from this study thus support the use of* Alchornea laxiflora* in folklore remedies for the treatment of infections caused by microorganisms.

The phytochemical analysis of the leaf extract of* A. laxiflora *revealed the presence of alkaloids, tannins, flavonoids, saponins, and reducing sugars. These compounds play important roles in bioactivity of medicinal plants; thus medicinal values of these plants rely on the embedded phytochemicals and as such produce definite physiological actions on human body. Flavonoid one of the phytochemicals resident in* A. laxiflora* leaf extract is known to play important roles in cleaning human bodies of oxides generated during metabolism.

Among the Yorubas of Southwestern Nigeria,* A. laxiflora* is an important plant for the treatment of stress, ageing, and other ailments. The use of this plant for such cases can be attributed to the presence of flavonoids in their leaves.

Further studies are ongoing in our laboratories on the mechanism(s) of actions of the extract on bacteria and fungi found to be susceptible to the effects of the plant extracts.

As far as we know, up to this point,* Alchornea laxiflora* leaf extract is a potent source of antibacterial and antifungal compound and compared favourably with the positive controls (streptomycin-antibacterial and Nystatin-antifungal) used in this study.

## Figures and Tables

**Table 1:** The sensitivity patterns, minimum inhibitory concentrations (MICs), and minimum bactericidal concentrations (MBCs) exhibited by *Alchornea laxiflora* crude leaf extract against tested bacterial isolates.

Bacterial isolates	Zones of inhibition (mm)^*^		(mm)^*^	
	*A. laxiflora * (25 mg/mL)	Streptomycin (1 mg/mL)	MIC (mg/mL)	MBC (mg/mL)
*Staphylococcus aureus* (NCIB 8588)	14 ± 0.00	20 ± 1.00	1.56	3.13
*Staphylococcus aureus* (SW)	19 ± 1.00	20 ± 0.50	1.56	3.13
*Staphylococcus aureus* (SW)	20 ± 0.00	20 ± 1.41	3.13	6.25
*Staphylococcus aureus* (SS)	18 ± 0.50	20 ± 1.41	3.13	6.25
*Staphylococcus aureus* (SS)	20 ± 0.00	20 ± 0.00	1.56	6.25
*Staphylococcus aureus* (SS)	22 ± 0.00	20 ± 1.41	1.56	3.13
*Staphylococcus aureus* (NC)	15 ± 0.00	20 ± 1.41	12.50	25.00
*Staphylococcus aureus* (NC)	17 ± 0.00	20 ± 0.50	3.13	12.50
*Staphylococcus aureus* (NC)	23 ± 0.50	20 ± 0.71	3.13	12.50
*Staphylococcus aureus* (NC)	14 ± 0.00	20 ± 0.00	6.25	12.50
*Micrococcus luteus* (NCIB 196)	15 ± 1.00	18 ± 0.71	1.56	6.25
*Pseudomonas fluorescens* (NCIB 3756)	12 ± 0.00	20 ± 0.00	1.56	3.13
*Bacillus cereus *(NCIB 6349)	23 ± 0.50	21 ± 1.22	3.13	6.25
*Clostridium sporogenes *(NCIB 532)	22 ± 1.00	24 ± 0.71	3.13	6.25
*Shigella *species (ST)	21 ± 0.50	11 ± 0.00	3.13	6.25
*Shigella *species (ST)	24 ± 0.50	11 ± 0.00	3.13	6.25
*Shigella *species (ST)	21 ± 1.00	11 ± 0.00	3.13	6.25
*Bacillus stearothermophilus *(NCIB 8222)	20 ± 0.00	21 ± 1.58	3.13	3.13
*Escherichia coli *(NCIB 86)	14 ± 0.50	11 ± 0.00	25.00	ND
*Escherichia coli *(ST)	21 ± 0.50	0	25.00	ND
*Escherichia coli *(ST)	14 ± 0.00	0	25.00	ND
*Escherichia coli *(ST)	20 ± 0.00	11 ± 0.71	25.00	ND
*Klebsiella pneumoniae* (NCIB 418)	14 ± 0.00	0	3.13	1.56
*Klebsiella pneumoniae* (SS)	14 ± 0.00	0	3.13	6.25
*Klebsiella pneumoniae* (SS)	16 ± 0.50	0	25.00	6.25
*Klebsiella pneumoniae* (SS)	16 ± 0.00	0	25.00	ND
*Bacillus subtilis *(NCIB 3610)	16 ± 1.00	19 ± 0.89	1.56	6.25
*Bacillus polymyxa *(ES)	15 ± 0.00	18 ± 0.00	0.78	1.56
*Clostridium pyogenes *(ES)	17 ± 1.00	20 ± 0.00	3.13	6.25
*Enterococcus faecalis* (NCIB 775)	21 ± 0.50	20 ± 0.00	6.25	12.50
*Proteus vulgaris *(ES)	14 ± 0.00	16 ± 1.41	1.56	6.25
*Pseudomonas aeruginosa *(ES)	16 ± 1.50	0	3.13	6.25
*Pseudomonas aeruginosa *(ES)	12 ± 0.00	0	12.50	25.00
*Pseudomonas aeruginosa *(ES)	16 ± 0.00	0	12.50	25.00
*Pseudomonas aeruginosa *(ES)	17 ± 0.50	0	12.50	ND
*Pseudomonas aeruginosa *(ES)	16 ± 0.50	0	12.50	ND
*Pseudomonas aeruginosa *(ES)	17 ± 0.00	0	25.00	ND
*Pseudomonas aeruginosa *(ES)	16 ± 0.00	0	12.50	25.00
*Bacillus anthracis *(ES)	17 ± 1.00	19 ± 0.71	6.25	25.00

NCIB: National Collection of Industrial Bacteriology; (mm)^*^: mean of three replicates; streptomycin: positive control; 0: no activity; ND: not determined; SW: surgical wound isolate; SS: sepsis wound isolate; NC: nasal cavity isolate; ST: stool isolate; ES: environmental isolate.

**Table 2:** The sensitivity patterns, minimum inhibitory concentrations (MICs), and minimum fungicidal concentrations (MFCs) exhibited by *Alchornea laxiflora* crude leaf extract against tested fungal isolates.

Fungal isolates	Zones of inhibition (mm)^*^		(mm)^*^	
	*A. laxiflora * (35 mg/mL)	Nystatin (1 mg/mL)	MIC (mg/mL)	MFC (mg/mL)
*Aspergillus niger *	22 ± 0.00	24 ± 1.55	35.00	ND
*Aspergillus fumigatus *	17 ± 0.50	25 ± 0.56	35.00	ND
*Aspergillus glaucus *	17 ± 0.10	22 ± 0.00	17.50	35.00
*Fusarium *species	0	17 ± 0.00	ND	ND
*Penicillium expansum *	12 ± 0.00	20 ± 0.55	17.50	35.00
*Alternaria *species	21 ± 0.50	16 ± 1.55	35.00	ND
*Trichophyton tonsurans *	23 ± 0.50	31 ± 2.00	2.19	8.75
*Trichophyton interdigitale *	17 ± 1.00	29 ± 2.00	17.50	35.00
*Penicillium camemberti *	17 ± 0.00	21 ± 1.00	17.50	35.00
*Trichophyton mentagrophytes *	23 ± 0.50	30 ± 1.00	17.50	35.00
*Trichoderma *species (nonpathogen)	13 ± 0.50	15 ± 0.00	8.75	17.50
*Aspergillus flavus *	16 ± 0.00	20 ± 1.55	35.00	ND
*Scopulariopsis brevicaulis *	18 ± 0.50	22 ± 1.22	35.00	ND
*Penicillium italicum *	14 ± 0.05	26 ± 1.52	8.75	35.00
*Trichophyton rubrum *	0	13 ± 1.50	ND	ND
*Candida albicans *	18 ± 0.50	28 ± 0.55	35.00	ND
*Candida pseudotropicalis *	18 ± 0.00	27 ± 1.51	8.75	17.50

(mm)^*^: mean of three replicates; MIC: minimum inhibitory concentration; MFC: minimum fungicidal concentration; 0: no activity; Nystatin: positive control; ND: not determined.

**Table 3:** The phytochemical analyses of *A. laxiflora *crude leaf extract.

Test	Results
Alkaloids	+
Tannins	+
Flavonoids	+
Saponins	+
Reducing sugars	+
